# Business Model Innovation Paths of Manufacturing Oriented towards Green Development in Digital Economy

**DOI:** 10.3390/ijerph192416454

**Published:** 2022-12-08

**Authors:** Xiao Han, Jiayun Zhang

**Affiliations:** School of Management, Wuhan University of Technology, Wuhan 430070, China

**Keywords:** green development, digital economy, manufacturing, business model innovation, proceduralized grounded theory

## Abstract

China’s manufacturing industry has been confronted with the issue of extensive development with high input, high consumption, and high emissions for a long time, and its green development is the key to reaching carbon neutrality in China. Under the digital economy, business model innovation is the fundamental means of the green development of manufacturing enterprises. Four representative listed companies in China’s manufacturing were selected as typical cases for the case study. Through open, axial, and selective coding that is based on proceduralized grounded theory, this study profoundly explores business model innovation paths of the manufacturing industry oriented towards green development in the digital economy following the research logic of “green development orientation–business model innovation process–business model innovation result”. Moreover, this study further compares the differences among paths and discusses each path’s effectiveness and applicable conditions. Results show that: (1) Four green business model innovation paths are revealed based on the four green development orientations: efficiency-oriented path, value-oriented path, user-oriented path, and ecology-oriented path. (2) Different enterprises pursue distinct business model innovation paths. The scientific premise for enterprises to opt for the optimal innovation path is the matching of upgrading demands, existing conditions, and path characteristics. Ultimately, the following policy implications are offered: First, promote the green innovation of business models in the manufacturing industry. Second, consider enterprises’ heterogeneity and implement differentiated support policies. This study can serve as theoretical support and decision-making reference for business model innovation and green development in manufacturing enterprises.

## 1. Introduction

**Figure 1:**
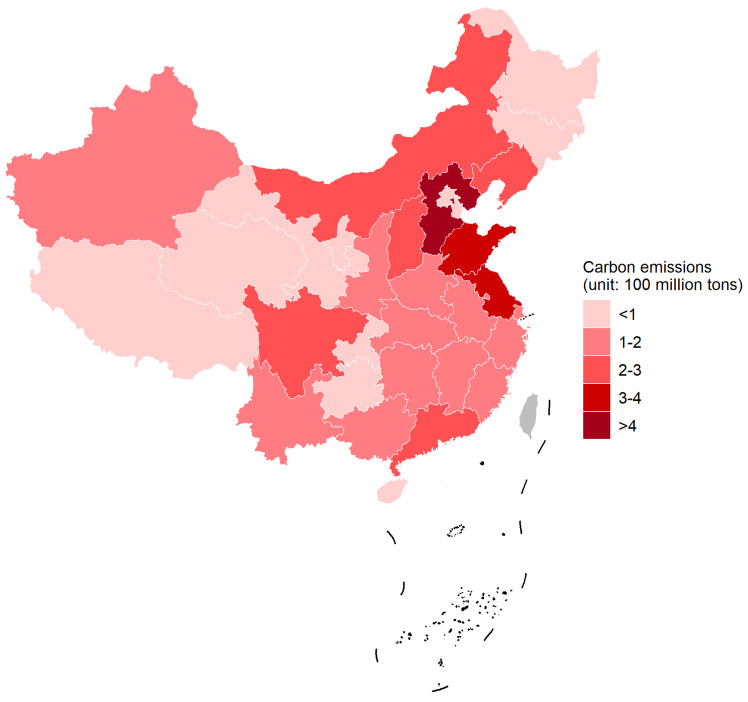
The industrial carbon emissions in the year 2021.

As a major carbon emitter, China is facing the dual pressure of low-carbon transition and an economic downturn brought on by the COVID-19 epidemic. Therefore, achieving green development—namely, innovation and development patterns that scientifically balance the ecological environment and economic growth—becomes a significant topic during the crisis [1,2]. As the “ballast stone” of China’s economy, the extensive development mode of the manufacturing industry has brought great pressure on the ecological environment. Therefore, the green development of the manufacturing industry has become the strategic focus of China’s continuous promotion [3,4]. As emphasized in the “14th Five-Year Plan for Industrial Green Development”, the next five years will be a critical period for implementing the strategy of manufacturing power and a crucial stage for the realization of green manufacturing. Hence, Chinese manufacturing enterprises need to accelerate their green transformation and improve their green innovation capability to drive green development [5,6]. However, from the perspective of industrial carbon emissions in 2021 provinces (as shown in Figure 1), the development form is still very severe. Faced with the requirements of green development, must manufacturing enterprises sacrifice their financial profits to fulfill their environmental obligations? Is there a win-win way to achieve both profit growth and green development? This issue remains to be explored. This paper studies business model innovation in manufacturing to integrate the environmental requirements in business activities fundamentally, and innovation practices seem necessary [7,8].

In recent years, China has gradually become an important leader and driver of the global digital economy [9]. Under the background of economic digital development and transformation, the integration of new-generation information technology and the manufacturing industry is able to empower all-around green business model innovation, including the improvement of value creation processes, the increase in the efficiency of value delivery, the expansion of value capture channels [10,11], and the promotion of the development of manufacturing industry to the direction of high-end, intelligent, green, and service [12]. This creates new opportunities for the green development of China’s manufacturing industry. However, the majority of studies on digital business model innovation in Chinese manufacturing enterprises have focused on maximizing corporate profits or performance [13]. Few studies have considered the value of digital business model innovation to the environment from the perspective of green development. Therefore, in the digital economy, integrating digital business model innovation with the green development of the manufacturing industry is a timely and important but not yet sufficiently explored research topic.

In general, existing studies on business model innovation in the academic circle have focused on the following. (1) they explore the influencing factors or driving forces of business model innovation based on the internal and external aspects of enterprises [14,15,16]. For example, Sorescu proposed that big data is able to drive and empower business model innovation to become a source of competitive advantage [17]. Yu et al. conducted multiple regression analyses on the data of 145 Chinese manufacturing enterprises to explore the impact of organizational search on business model innovation [18]. (2) they explore the types of business model innovation based on different dimensions [19,20,21,22]. For example, Sun et al. selected three representative enterprises for their case studies and classified sharing economy business models into nine basic types based on two dimensions, namely, thematic type and manifestation [23]. Duparc et al. obtained seven types of open-source business model innovation through a structured review of the literature and cluster analysis of 120 case data [24]. (3) they explored the performance impact and evaluation of business model innovation [25,26,27,28]. For example, Guo et al. explored the contribution of the three elements of business model innovation of the enterprise to the performance of digital innovation from a demand-side perspective [29]. Bockin introduced business model lifecycle assessment, which refers to a quantitative method for evaluating and comparing the environmental performance of the business model [30].

The aforementioned research perspective has formed diverse valuable research results. However, three deficiencies remain. First, existing studies pay more attention to the influencing factors and evaluation of business model innovation [31,32,33], while few researches discuss the evolution and development of the enterprise business model from the perspective of process, especially the induction and summary of the innovation path of the enterprise business model. Second, the present research lacks grounded case studies on the innovation practices of China’s successful manufacturing enterprises in the context of the digital economy and green development [34,35], and few studies can provide practical direction for enterprise digital and green business model innovation. Third, existing research on the innovation of the manufacturing business model is mostly a single case study [36,37,38] that lacks comparative research among different enterprises. However, as China’s manufacturing industry has large volumes and diverse categories, it is necessary to use multi-case studies on this basis to improve the research’s universality and explore differentiated business model innovation models and paths of manufacturing.

Based on the above analysis, this study takes a number of typical Chinese manufacturing enterprises as the research object and selects the green development pioneers who embedded digital technology into their business model for green business model innovation as samples. This study systematically analyzes business model innovation paths of manufacturing oriented towards green development of manufacturing under a digital economy through multi-case research and three-level coding and additionally performs a comparative study on the paths. This paper aims to fill the existing research gaps, enrich the business model innovation and green development theory, and serve as practical guidance and theoretical reference for manufacturing enterprises and the government.

This study is organized as follows. Section 2 provides a detailed review of the literature and research framework. Section 3 details the research design of this paper, including the research methods, study case selection, data collection, and data coding process. Section 4 presents the result analysis, which introduces the four paths revealed by coding in detail, constructs their conceptual models, and puts forward four propositions. Section 5 involves a discussion that compares the four paths, discussing the heterogeneity among paths and also pointing out the efficiency and the applicable condition of each path. Section 6 discusses the main conclusion. Section 7 discusses theoretical contribution, management implications, and policy implications. Section 8 describes the limitations and prospects of this study.

## 2. Literature Review and Research Framework

### 2.1. Literature Review

With regard to the so-called enterprise business model, scholars have conducted research in light of value chains, enterprise systems, organizational management, and the science of strategy [39,40,41,42], which have essentially identified the business model as an architecture of activities that create, deliver and capture value in an enterprise [43]. Business model innovation is a process of organizational transformation in which organizations seek to create, deliver, and acquire value for stakeholders through new value propositions [44]. It is a new method and logic for enterprises to explore value creation, as well as a crucial strategy for enterprises to develop competitive advantages [45].

The digital economy is the increasing application and integration of digital technology in the whole economy and society [46]. Digital technology provides a new perspective and way for enterprises to discover and create value and becomes the entry point of business model innovation for today’s enterprises [47,48]. Existing studies have pointed out that business model innovation in the digital economy depends on the fundamental components of digital transformation, including the design of digital products or services and digital platforms, which stand for the results of the combination of digital technology elements [49]. On the one hand, digital products or services are supported by digital technology and are products or specific service solutions with perceptual interaction and iteration functions [50,51]. On the other hand, a digital platform is a major form in which digital technology plays a role in the transformation of enterprise business form [52,53]. Due to the characteristics of emerging technologies in the digital economy, the research on business model innovation in the context of China mainly focuses on the Internet and e-commerce [54,55]. For example, based on the perspective of strategy and resources, Wang Bingcheng took Internet service enterprises as research samples, built the driving mechanism model of business model innovation of Internet service enterprises, and revealed three modes to achieve business model innovation [56]. The current exploration of manufacturing is relatively small. As a supporting industry of China’s national economy, the manufacturing industry has not been fundamentally changed in the situation of being large but not strong, complete but not excellent [57].

Business model innovation is an important “window of opportunity” for our manufacturing industry transformation and upgrading [58]. Under the digital economy, manufacturing business model innovation should pay attention to not only economic value but also environmental value [59,60]. With the increasing importance of green development, scholars have been widely concerned about how to achieve green development [61,62,63]. Recent studies have found that business model innovation can promote sustainable development and mitigate the associated negative environmental externalities [63,64,65]. For example, the leasing and sharing model can encourage enterprises to design products around durability and improved quality, make remanufacturing feasible, and reduce the total production of products and the demand for resources [66]. There is also the product service model, which provides value-added services for the product life cycle, separates value creation from material and energy consumption, and significantly reduces the impact on the environment compared with a pure product system [67]. In addition, some scholars point out that different upgrading pressures faced by enterprises will promote the formation of different green development orientations and then affect various types of performance with differentiated innovation paths [68,69].

To sum up, although digit and green have become two important directions of manufacturing enterprises’ business model innovation, academic research is still lacking. In essence, the premise of green development is sustainable economic development. We must innovate the business model, balance the relationship between green development and enterprise profit, and unify ecology and marketization in order to realize green development truly. Most of the existing research only explores the business model innovation of Chinese manufacturing enterprises from the perspective of higher competitive advantages and enterprise performance, ignoring the combination of China’s current green development situation. It is necessary to extend the connotation of green development in business model innovation, seek a business model innovation path that takes into account enterprises’ profit growth and green development, and guide the green transformation of manufacturing enterprises. These are the core issues to be solved in this study.

### 2.2. Research Framework

**Figure 2:**
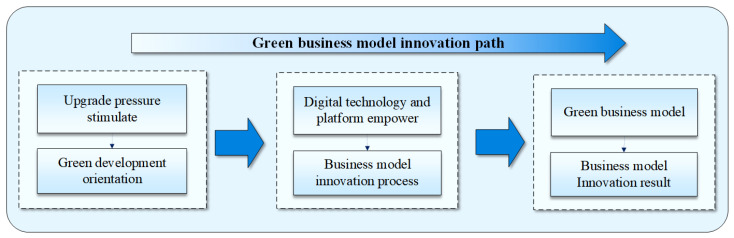
Research framework.

Through the above review of the literature, this study intends to consider that the business model innovation process oriented towards green development in the digital economy is as follows: On the basis of the green development orientation that corresponds to the upgrading pressure, manufacturing enterprises utilize digital technology and digital platforms to innovate the logic of original value creation and to achieve green business model innovation finally. Based on this process, to further explore the specific green business model innovation paths of manufacturing, whether differences exist between these paths, and what application conditions are required for each path, this paper constructs an analytical framework based on the basic logic of “green development orientation–business model innovation process–business model innovation result” (as shown in Figure 2). This paper uses the case study method to analyze the green development orientations, business model innovation process, and business model innovation results of a number of typical Chinese manufacturing enterprises systematically and examines and compares their green business model innovation paths. This work aims to make up for the shortcomings of existing research and achieve the effective expansion of theory.

## 3. Research Design

### 3.1. Research Method

This paper uses grounded theory to conduct the exploratory multi-case study, primarily for the following reasons. (1) The purpose of this study is to analyze the business model path for green development under the digital economy, which belongs to the “how—question”. Exploratory case analysis lies in the “discovery logic” rather than the “verification logic” [70], which can clearly explain the “how-to problem” and is very consistent with the research theme. (2) the business model innovation is dynamic and complex. The grounded case study can repeatedly focus and comparison based on textual data to further study the business model innovation experience of sample enterprises so as to reveal the business model innovation path and unleash the advantages of theoretical construction based on “Phenomenon Driven” [71] (3) Compared with the limitations of single case studies, multi-case studies can help identify and compare the similarities and differences among different types of business model innovation, which improve the external validity of the research, enhance the persuasiveness and universality of the conclusion and are more in line with the replication logic of the case study. (4) Procedural grounded theory provides strict criteria, steps, and procedures for qualitative analysis, and its rigorous techniques and methods enable the research to carry out process tracing and repeated testing, making up for the defects of general qualitative methods that the process cannot be traced and the conclusion is difficult to test. It includes open coding, spindle coding, selective coding, and theoretical saturation test. This highly systematic data selection and analysis procedure will be strictly followed in this paper to improve the accuracy, rigor, and verifiability of research findings.

### 3.2. Research Case Selection

The sample selection for the case study is based on the following considerations. (1) Universality and heterogeneity of cases. The issue of the business model innovation path for enterprises requires cases with widespread or variable consumer types, production characteristics, and establishment periods. (2) Availability and adequacy of data. The annual reports and other public information of four companies listed as case companies are available. Their development model has also captured widespread attention from the media and research institutions, and a large amount of news, the literature, and additional relevant information has been published. (3) Representativeness and typicality. All four companies exhibit strong industry representation and leadership. Hence, they are robust benchmarks in their fields. Moreover, the case companies have a good demonstration role in the green development of China’s manufacturing industry. All four companies fully implement the national green and low-carbon development strategy, adhere to green innovation to help the development of companies, and achieve the coordinated development of economic and environmental benefits. (4) The principle of the matching between the case object and the research questions. Samples were selected to fit the themes of the “digital economy and green development” and “business model innovation.” The case enterprise started the digital transformation earlier, and on this basis, it has successfully implemented the green business model innovation to achieve green development and thus has rich experience in innovation and development. In summary, the case enterprise information is as Table 1.

In order to improve the reliability of the research, according to the idea of continuous analysis of grounded theory, the case enterprises are divided into a modeling group and a testing group. In accordance with the principles of the availability of information, the typicality and heterogeneity of the selected cases, and the matching of the research problems, five cases of Changan Automobile Group, Shenyang Machine Tool, Xi’an Shaangu Power, Qingdao Red Collar Group and Haier Group were selected to test the saturation of the theoretical model.

### 3.3. Data Collection

As a representative enterprise in its field, the growth and innovation mode of Xiaomi Corporation, Goldwind Company, Qumei Group, and Baosteel Group have captured widespread attention in the industry and academia, thereby making various publicly available interviews, surveys, books, reports, and other materials abundant to cover research needs sufficiently. The specific sources of the case materials in this paper as Table 2. Multi-channel information sources help avoid homologous bias and enhance the “construct validity” of the case study. In order to ensure the authenticity and reliability of the case data, the data acquisition process conforms to the “triangulation verification” principle. Finally, the case document library is constructed according to the collected data to provide the basis for coding analysis so as to improve the reliability of the case study.

### 3.4. Data Coding

In this study, data coding is carried out according to programmed grounded theory. In order to avoid the influence of the coders’ individual subjectivity, reduce the errors in the case study and improve the sensitivity of the theory, this study formed a coding team to complete the coding process, and the members adopted the form of “back-to-back” independent coding analysis, comparison and discussion until a consensus was reached. The coding process is as follows, open coding, spindle coding, selective coding, and theoretical saturation testing.

#### 3.4.1. Open Coding

Open coding involves repeatedly refining the original material, extracting information regarding the research object, and conceptualizing and classifying it. This paper focuses on the “Business model innovation of manufacturing enterprises orientated to green development under the digital economy” to conduct open coding. First, the words and sentences related to this content in the material are marked and preliminarily simplified into concepts. This step is followed by “categorization,” in which concepts that seem related to the same phenomenon are grouped into subcategories. The final four cases resulted in 61 subcategories (as shown in Table 3).

#### 3.4.2. Axial Coding

Axial coding, which is based on open coding, seeks the organic connection between concepts. Its primary task is to find and establish various associations between conceptual categories. Based on the research framework of this paper, second-level coding summarizes and deduces the concepts of green development orientation and business model innovation behavior involved in first-level coding further. For example, the five pairs of “Introduction of automated equipment,” “standardization of operation process,” “basic data collection,” “data integration,” and “production automation” in the first-level coding can be integrated into an axis: The enterprise introduces automated equipment, accesses the Internet for the basic data collection on production equipment, and automates the production process by integrating data through standardized operational processes to form automated production lines for systematic and continuous production. Therefore, these five subcategories can be incorporated into the main category of “production process automation.” Through axial coding, 17 main categories are extracted (as shown in Table 4).

#### 3.4.3. Selective Coding

**Figure 3:**
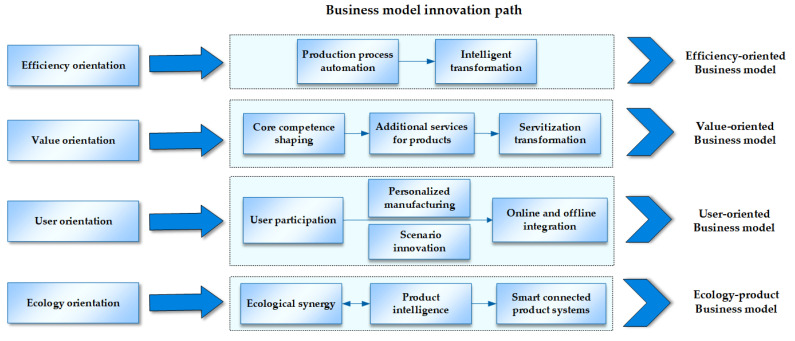
Research framework.

Selective coding is a three-level coding in proceduralized grounded theory, which determines the core category after a systematic analysis of the concept categories found. Subsequently, the core category and other categories are systematically integrated to form a “storyline.” Further, through the interaction between materials and emerging theories, the categories and their relationships are continuously improved to construct a theoretical model(as shown in Figure 3). This paper takes the green development orientation as the core category and forms a storyline through the matching of green development orientation and business model innovation behavior, as well as the matching between innovation behavior, to construct the path model. Three-level coding results were obtained on the basis of the analysis and comparison of case data, concepts, and categories. Through selective coding, four paths are extracted (as shown in Table 5).

#### 3.4.4. Theoretical Saturation Test

The grounded theory method requires researchers to constantly collect and analyze data and constantly supplement and improve emerging concepts and categories. When the newly collected data fails to produce new categories and relations, it indicates that the theory has reached saturation. In order to test whether theoretical saturation has been achieved, this study grounded the cases of Changan Automobile, Shenyang Machine Tool, Shaan-Gu Power, Qingdao Red Collar, and Haier in the test group according to the same method. The encoding results are shown in Table 6. Although some new concepts have been separated out in this coding, these new concepts can be included in the categories of the above analysis, and no new categories and structural relations have been found. It shows that the coding results of this study have good theoretical saturation and validity.

## 4. Case Analysis

This paper uses grounded theory and multi-case study methods to systematically analyze the green development orientation and business model innovation behavior in different case situations and then construct the efficiency-oriented path, value-oriented path, user-oriented path, and ecology-oriented path. The four green business model innovation paths are discussed in detail, from the green development orientation, business model innovation process, and the business model innovation result.

### 4.1. Efficiency-Oriented Path: From Production Process Automation to Intelligent Transformation

**Figure 4:**
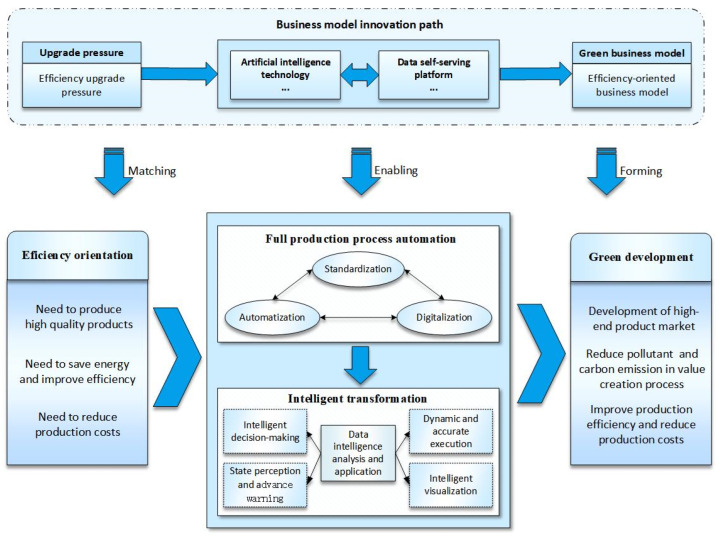
Conceptual model of the efficiency-oriented path.

Enterprises are under pressure to upgrade their efficiency, accordingly taking efficiency improvement (i.e., producing high-quality products, saving energy, improving efficiency, and reducing production costs) as the green development orientation. Based on this orientation, the efficiency-oriented path is meant for enterprises to achieve intelligent transformation through the gradual automation and digitization of the production process to resolve efficiency issues and enhance product quality. Efficiency-oriented enterprises implement automation equipment, accelerate digital construction, and connect diverse plant equipment to the Internet for data collection. These measures make the advantages of automated manufacturing superimposed with networking and digitization, give full play to the role of digital technology in controlling and enabling industrial development, and implement the automation of the production process. Furthermore, enterprises transform into intelligence, reconfigure critical resources, and optimize specific processes. Based on the data self-service platform to achieve intelligent analysis and application of production data, such as state perception, intelligent decision-making, advanced warning, and intelligent visualization. This path ultimately achieves an upgrade in the efficiency of the whole industrial chain under upgrading technology levels [72], reduces pollution emissions, improves quality and efficiency, and maximizes energy and resource utilization (as shown in Figure 4).

This path has two key nodes. (1) Automation of production processes. Enterprises effectively integrate production lines, processes, and procedures and use automation technologies and artificial intelligence to establish an automated production line group that runs through the whole process from raw materials into the factory to finished products. To optimize its production process, the Baosteel Group executes the “machine substitution” strategy and introduces new technologies, such as industrial robots, unmanned cranes, and artificial intelligence. On the one hand, it has developed a unified standard for operation mode, operation process, and information processing to achieve the acquisition of product lifecycle data. On the other hand, it unifies data structure so that the upper and lower process data in the workshop can flow and cooperate and thus implement the full automation of the production process gradually. Based on this, the automation level of the Baosteel Group has reached a new height and is close to the advanced international level, making the production process more safe and efficient and realizing the improvement of the production line quality index, cost index, and energy consumption index. This indicates that manufacturing enterprises should carry out the automatic transformation on the basis of the standardization and standardization of their production process so as to improve the working efficiency and solve the problem of industrial manufacturing safety production.

(2) Intelligent transformation. Together, intelligent machines and human experts form a human–machine integrated intelligent system, which will drive manufacturing development to a new level. During the product process, the system will carry on independent analysis, reasoning, and decision-making for the simulation of the process. It continuously improves its own intelligence through data accumulation, thereby providing strong support for energy conservation and environmental protection, product quality improvement, cost control, and efficiency enhancement. Intelligence is the direction of development for manufacturing automation, which updates the concept of manufacturing automation and extends it to flexibility, intelligence, and a high degree of integration. The Baosteel Group is currently in this process, and the main action strategy is to establish a human–machine integrated intelligent system and implement intelligent analysis and application of data continuously. As an example, the development of an energy efficiency diagnostic model for steel furnaces, the online, real-time monitoring of pollution factors, and the comparison of energy consumption and pollutant emissions with standard lines for early warning in order to maximize energy efficiency and minimize pollutant emissions. In addition, a higher level of intelligence is also available in the form of virtual reality platforms. This application simulates the performance of products, equipment, and plants in real-life situations, thereby enabling enterprises to inspect in a virtual environment before proceeding into production and then optimizing the entire process in parallel based on the test results to reduce resource waste and environmental pollution. This manifests that enterprises should take digital transformation as the starting point to complete the construction of a series of platforms and systems, such as an integrated control platform, AR/VR system, integrated decision IOC and intelligent diagnosis to strengthen their ability to intelligent data analysis, and build visual, transparent, and intelligent manufacturing.

Based on the above analysis and discussion, combined with coding materials, this paper proposes Proposition 1:

**Proposition 1.**  *Based on efficiency orientation, production process automation and intelligent manufacturing represent an important green business model innovation trend. Manufacturing enterprises digitalize and automate the production process by introducing digital technology and implementing autonomous control and dynamic production to optimize production and reduce consumption and emissions. Then, they further analyze and apply the production data intelligently to achieve intelligent transformation, improving the accuracy of decision-making to save energy, reduce emissions and enhance the quality of products. Such innovation increases the efficiency of resource utilization in the production process and reduces environmental pollution so as to promote the green development of manufacturing.*

### 4.2. Value-Oriented Path: From Core Competence Shaping to Servitization Transformation

**Figure 5:**
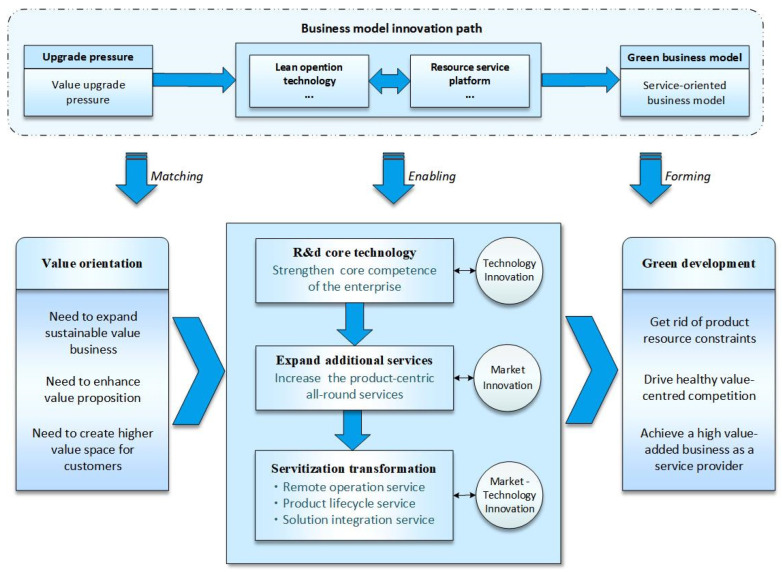
Conceptual model of the value-oriented path.

Enterprises are under pressure to upgrade their value proposition, accordingly taking value enhancement (i.e., expanding sustainable value business, enhancing value proposition, creating higher value space for customers) as the green development orientation. Based on this orientation, the value-oriented path is meant for enterprises to transform from merely supplying products to supplying additional services for products, which eventually provide total solution services to achieve servitization transformation. Value-oriented enterprises primarily reinforce digital technology research and development and patent construction to shape the unique core capabilities (technological innovation) constantly. With their core capabilities, they further explore high-value-added markets (market innovation) and provide additional services for products. Lastly, enterprises are capable of developing resource service platforms to rapidly supply digital services [73], such as intelligent operation services and total solution services, so as to achieve the servitization transformation from product manufacturer to the solution service provider (technology-market innovation). This path ultimately shapes the advantages of sustainable development of enterprises, eliminates product resource limitation through servitization, and leads the healthy industry competition driven by technology and service innovation (as shown in Figure 5).

This path has three key nodes. (1) Core competence shaping. Enterprises continue to develop critical core technologies to shape a unique competitive advantage. With a focus on technological innovation, enterprises shape the ability to increase value for customers, thereby laying the foundation for subsequent servitization. For instance, the Goldwind Company continued to invest in scientific research over the years and insisted on independent research and development and innovation of wind power technology, which laid a leading edge for its long-term sustainable development. As the technology continues to break through, the technology level and products of Goldwind Company have also taken a leading position in the world. Digital technology runs through their industrial chain and has intelligent operation and management capabilities for labor scheduling, cost and risk control, energy efficiency prediction, asset management, and economical operation, forming the core advantages and competitiveness of the enterprise. This indicates that the premise of enterprise servitization is to develop distinctive core competence of delivering irreplaceable services.

(2) Additional services for products. To change the homogenization of market products and the continuous decline of core business profits, enterprises desire to expand various payment services while selling products and achieve the service of core technology. Accordingly, enterprises adopt sustainable considerations to address customer needs, broaden their business scope, and provide professional services supported by core technologies. For example, the Goldwind Company provides maintenance, wind turbine design, old equipment technology system upgrade, and other services. At this time, the service model of Goldwind Company mainly provides product-centric services to increase the added value of products and accumulate technical service experience and related markets.

(3) Servitization transformation, from manufacturer to servitization provider. The development of servitization in manufacturing aims to generate value-added and create sustainable value. Providing total solutions meet this demand. As a result, manufacturing enterprises further improve the degree of service, that is, from providing additional services for products to providing integrated to meet the value of customers at a high level of the total solution services. In the course of its development, the Goldwind Company has invariably insisted on becoming the “leader” of integrated wind power solutions that strives to create greater value for users in all aspects of wind power development and operation. Therefore, Goldwind deepens the intelligent and digital operation and maintenance into the whole value chain of operation and maintenance services, providing customers with comprehensive digital and high-level solution services instead of homogenized and replaceable services. For instance, the Goldwind Company created the online Smart Operation System- SOAM™, which integrates unified IT tools with advanced applications, such as centralized power prediction, intelligent fault diagnosis, and health status warning, and provides customers with professional and convenient integrated energy optimization solutions. With the servitization transformation, Goldwind’s main business has shifted to providing functions rather than ownership, identifying business areas with long-term value, and helping other related enterprises to optimize their energy operations and achieve energy saving and emission reduction. This proves that servitization transformation can lead to the green development of the entire upstream and downstream industry chain, co-establish a high-quality ecosystem, and mitigate social and environmental risks.

Based on the above analysis and discussion, combined with coding materials, this paper proposes Proposition 2:

**Proposition 2.**  *Based on value orientation, providing total solutions and developing platform-based digital services represent a significant green business model innovation trend. Manufacturing enterprises independently R&D critical core technologies to expand their service businesses with unique competence. Afterward, through the digital platform, they integrate various resources to form a resource service platform, provide integrated digital solutions, help customers expand their value space, and achieve service transformation. Such innovation enhances the value proposition of enterprises by identifying the business areas with long-term value and leads the entire industry to create a higher level of sustainable value so as to promote the green development of the manufacturing industry.*

### 4.3. User-Oriented Path: From Personalized Customization to Scenario Innovation

**Figure 6:**
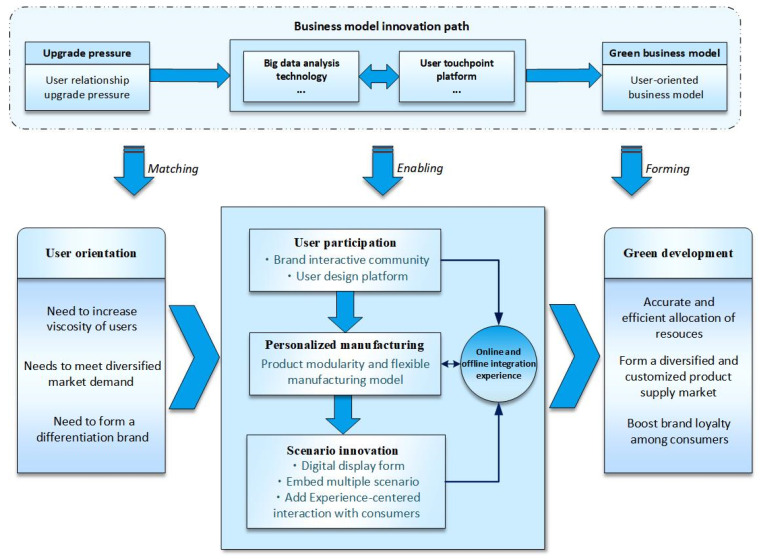
Conceptual model of the user-oriented path.

Enterprises are under pressure to upgrade their user relationships, accordingly taking user relationship enhancement (i.e., increasing the viscosity of users, meeting diversified market demands, forming a differentiation brand) as the green development orientation. Based on this orientation, the user-oriented path is meant for enterprises to entitle users to participate in the manufacturing, implement personalized customization based on agile manufacturing, emphasize experiential user consumption, and shape a differentiated brand image with scenario-based innovation. On the one hand, user-oriented enterprises’ manufacturing and delivery must acquire and satisfy the preferences of consumers; on the other hand, they must produce economically and sustainably to achieve rapid integration and timely response of resources. With the support of digital technology, the enterprise online, through the construction of a user touchpoint platform, gains a comprehensive insight into consumer demands, generates user participation and design, and uses big data analysis to capture personalized market demands and achieve an accurate allocation of resources. Moreover, enterprises offline implement scenario innovation, embed multi-scenario elements to promote experiential consumption, and further strengthen the interaction between enterprise and consumers online and offline to shape a brand concept that is in tune with the spirit of the audience. This path ultimately shortens the distance between enterprises and consumers, improves the relationship between enterprises and consumers, and achieves accurate resource allocation to prevent resource mismatch and resource backlog [74,75] (As shown in Figure 6).

This path has three significant nodes: (1) To achieve customized production. Consumers have tended to pursue personalized spiritual satisfaction in recent years. At the same time, digital technology reduces conversion expenses and substantially affects the stability and sustainability of client resources. Therefore, enterprises should implement a user-centered pattern to develop their sustainable competitive advantage and reduce waste triggered by the misallocation of resources. For example, using the “DESIGN IN” model through the user design platform or brand interactive community on the demand side, consumers can participate in the product design process, alleviating market information asymmetry and reducing communication costs. This model meets users’ individual needs, facilitates word-of-mouth communication, and contributes to the development of corporate brands. It is worth noting that personalized customization necessitates that manufacturing enterprises increase their market adaptability to shorten the product development cycle and eliminate inventory. Consequently, flexible manufacturing has become an inevitable choice for manufacturing enterprises to improve the dynamic adaptation between product supply and demand. For instance, Qumei Group has improved its manufacturing level on the supply side through digital management and modular production, increased the flexible delivery capacity of personalized consumption trends, and captured the forward-looking market. In short, the original homogeneous product competition is not conducive to economic growth and results in a waste of resources, so enterprises should use digital technology to shorten the product manufacturing cycle and enhance the rapid response capability of manufacturing so that personalized and flexible production becomes an essential strategy at the enterprise production level.

(2) Scenario innovation. Offline scenario innovation is reflected in three touchpoints: digital touchpoint (digital display forms and carriers), physical touchpoint (traditional single scenario to multi-scene synthesis), and interpersonal touchpoint (add experience-centered interaction with consumers). Using “You + Living Hall” of Qumei Group as an example, the experiential consumption of leisure, entertainment, and shopping is realized through the home scenario layout, and it further accomplishes the visceral interaction between consumers. It also incorporates digital technologies such as virtual reality and artificial intelligence, enabling consumers to perceive information online while enhancing the consumer experience and promoting business sustainability. This implies that guided by data and taking experience as the starting point, Qumei Group has added more products and consumption scenarios around users’ needs, gradually connecting the links of users’ lives with each Qumei scenario and establishing a strong connection. In conclusion, in the current era of green development, the products and services produced must have not only economic value but also spiritual cores, such as experience and emotional value. As a result, enterprises should adapt to the live scene, integrate scientific and technological elements, and highlight the comprehensive experience of consumers through cross-border industrial integration, so as to actualize the value resonance with consumers.

(3) Combining offline and online innovation. The traditional storefront model is being replaced by online and offline commerce integration. The advantages of online commerce integration include commercial flow, information flow, and capital flow, which can reduce intermediate links and provide enterprises with timely access to data and information to optimize the allocation of resources. Offline commerce integration has advantages in logistics, service, and experience, and the arrangement of offline scenarios is crucial for stimulating consumer demand, increasing user experience, and fostering consumer loyalty. By combining the benefits of online business flow, information flow, and capital flow with the benefits of offline logistics, service, and experience, the advantages can be amplified geometrically, bringing greater benefits to the economy, society, and the environment.

Based on the above analysis and discussion, combined with coding materials, this paper proposes Proposition 3:

**Proposition 3.**  *Based on user orientation, product customization and experience-centered scenario innovation represent a significant green business model innovation trend. Manufacturing enterprises leverage digital platforms to engage users in manufacturing, utilize big data to excavate the user’s expectations, and enhance manufacturing responsiveness, so as to meet the individualized needs of users. Then, they develop experiential consumption through scenario innovations such as cross-border integration and digitalization of shops, and finally, achieve integrated development online and offline. Such innovation ameliorates the market situation of a large number of homogenized products, increases the viscosity of users, and achieves accurate allocation of resources to reduce resource wastes so as to promote the green development of manufacturing*.

### 4.4. Ecology-Oriented Path: From Product Intelligence to Smart Connected Product Systems Construction

**Figure 7:**
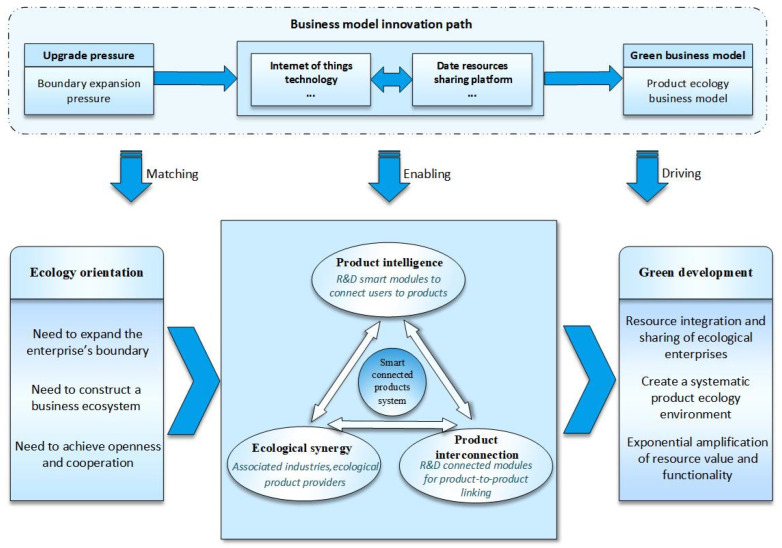
Conceptual model of the ecology-oriented path.

Enterprises are under pressure to expand their boundaries, accordingly taking ecological synergy (i.e., expanding the boundary of the enterprise, forming a business ecosystem, and achieving openness and cooperation) as the green development orientation. Based on this orientation, the ecology-oriented path is meant for enterprises to produce smart connected products, achieve cross-departmental collaboration by constructing a business ecosystem to enrich enterprise product categories, and finally establish smart connected product systems by ecological platforms. Ecology-oriented enterprises develop information resources sharing platforms and construct a business ecosystem comprised of associated industries and accessory product suppliers in a nested, value-added manner to diversify their product offerings, increase their control over pivotal resources, and expand their boundaries. Afterward, through large-scale and highly integrated connections between intelligent products, the product and business scope of enterprises is extended to a set of associated smart and connected scenario products and services. Furthermore, through multi-scenario linkage, the intelligent connection of all things is realized. This path ultimately achieves resource connection and sharing with external stakeholders, interconnects previously isolated resources to perform well together as a system, and generates considerably better resource value and functionality [76] (As shown in Figure 7).

This path has two significant nodes: (1) Product intelligence. Enterprises enhance the digital and intelligent levels of their products to make them more user-friendly and expand their functionality and value. By embedding different IT technologies (e.g., software), physical products transform into intelligent products that comprise hardware, sensors, and communication components. Intelligent products enable identifying user behavior and generating user behavior data and utilize intelligent computing capabilities (e.g., edge computing) to record, calculate, and even think autonomously. For instance, the acceleration sensor in the bracelet of Xiaomi Corporation counts steps by measuring the amount of change in direction and acceleration. Then, the smart module in the bracelet processes the data through intelligent computing capabilities to match the exercise types of users and supply a more scientific exercise program for the user. This predicts that product intelligence is a development trend for the transformation and upgrading of traditional manufacturing industries, such as mobile phones, wearable devices, and home appliances. Through the development of digital technologies such as the Internet of Things (IoT), enterprises can optimize the original functions of products, strengthen the connection between products and users to improve the added value of products, and increase their market control.

(2) Construction of the smart connected product systems by the ecological platform. In the process of smart product development, enterprises use a unified IoT smart chip to promote the generation of standardized data to determine the interactive linkage of products so as to create a systematic product ecological environment for users. For instance, Xiaomi Corporation has launched a smart home system: based on PM2.5, PM10, CO_2_, temperature, and humidity detected by the air detector, and the system creates a comfortable indoor environment by linking air conditioners, air purifiers, and humidifiers to form a new systematic function. This indicates that the smart-connected components of smart-connected products not only optimize the product functions but also integrate individual, discrete products into customized, integrated system solutions to meet the broader potential needs of users. The smart connected product is no longer an entity with a single function but a platform bearing multiple functional modules, which constantly reshapes the internal product boundary and expands the enterprise boundary through product interactive connection.

However, due to the diversification of consumer demands, the resources of an individual enterprise often fail to meet the construction of smart connected product systems through manufacturing or purchasing. Enterprises usually pursue a business ecosystem of cross-sectoral collaboration to share resources, knowledge, wealth, and value creation and achieve the synergy of ecosystem resources. For instance, the Xiaomi Corporation has launched the Xiaomi Eco Cloud, which enables data interaction and sharing among different products. Other enterprises can access their clouds to the Xiaomi loT platform through the Eco Cloud and incorporate it into the “Mi Home App” to achieve unified control so that their resources and external resources can implement synergetic development. This approach consolidates information from different products and enterprises across organizational boundaries to further increase the value of data reuse and amplify the role of resources. In addition, data sharing based on a cloud platform will not significantly increase the cost of organization operation, which can effectively avoid the internal production cost of organizations will continue to increase with the expansion of borders. This reveals that enterprises are able to take advantage of the general ecological platform as the data path points to achieve product links and data sharing. By constructing cross-industry and cross-field hyperlinks, the integrated communication and collaborative utilization of multi-source heterogeneous information flow are able to be actualized so as to establish a large-scale, cross-boundary, hyperlink, and highly integrated production system.

Based on the above analysis and discussion, combined with coding materials, this paper proposes Proposition 4:

**Proposition 4.**  *Based on ecology orientation, Providing smart connected products and building business ecosystems represents a significant green business model innovation trend. Manufacturing enterprises use digital technologies, such as the IoT, to generate the intelligence and interconnection of products and improve their added value. Afterward, by building a generally shared platform to achieve a business ecosystem across the organizational boundaries of multiple stakeholders, they gradually construct smart connected product systems to form intelligent life scenario products, such as smart homes. Such innovation optimizes product attributes and creates a business ecosystem to generate large-scale, cross-border collaborative innovation and data sharing, making the resource create higher value and function so as to promote the green development of the manufacturing industry.*

## 5. Comparison of Four Business Model Innovation Paths

The so-called business model innovation path is a general term for the direction, starting point, focus, process, and methodological means to achieve business model innovation. The coding process and the explanatory argumentation of typical cases take the basic elements of the business model innovation path as the main line of research and analysis and the focus of the investigation. Therefore, the four business model innovation paths in the previous section are compared comprehensively around the essential elements of the paths, such as the starting point, the direction and focus, the innovation process, effectiveness, innovation risk, and applicable condition (as Table 7).

The comparison between the paths and the case studies of the four manufacturing enterprises indicates that differentiated optimal innovation paths exist for various enterprises. First, around the critical aspects of innovation, such as production processes, technology development, market response, and product development, enterprises select their innovation starting point based on upgrading needs. Second, innovation focus and innovation process differ significantly between paths, thereby further resulting in varying effectiveness and applicable condition. Therefore, enterprises should combine their existing resources and capabilities to achieve an optimal match with differentiated innovation paths in accordance with the upgrading pressures they face.

## 6. Conclusions

This paper has performed an exploratory study by means of a multi-case study method and the encoding technique of proceduralized grounded theory. The main research findings are presented as follows.

(1) Based on the green development orientation, the four green business model innovation paths, including the efficiency-oriented path, value-oriented path, user-oriented path, and ecology-oriented path, are revealed. They utilized digital technology to change the content and logic of the original business model of enterprises, implement tangible value appreciation or intangible value development, and create economic and environmentally sustainable value. Specifically, the efficiency-oriented path leans toward efficiency improvement, which maximizes the efficiency of energy and resource utilization and minimizes pollutant emissions in the production process by transforming the production process from automation to intelligence and reducing production costs. The value-oriented path leans toward value enhancement, which enables enterprises to reposition themselves in the industry or value network with servitization transformation, develop digital services to break away from the contradiction between business growth and resource overload, and lead the sustainable development of the industry. The user-oriented path leans toward the user relationship deepening, which creates a locked-in user effect with personalized customization and scenario-based innovation to eliminate homogeneous competition in the industry, thereby increasing the viscosity of users and achieving precise resource allocation to prevent resource mismatch and waste. The ecology-oriented path leans toward ecological synergy, which constructs smart connected product systems with a business ecosystem of cross-department collaboration through a digital platform, and expands enterprise boundaries, thereby maximizing the resource portfolio value.

(2) With regard to the basic elements of the paths, such as the starting point, the direction and focus, the innovation process, effectiveness, innovation risk, and applicable condition, this paper compares the four green business model innovation paths comprehensively, identifies the differences among the four paths, and discusses the effectiveness and applicable condition of each path. Further analysis demonstrates that the business model innovation path has differentiated characteristics, and the optimal business model innovation path of an enterprise is determined by the matching relationship among innovation path characteristics. Their business model upgrade demands and their existing conditions.

## 7. Contributions of the Study

### 7.1. Theoretical Contributions

The research in this paper has enabled a richer research perspective on the fundamental path and path selection of business model innovation oriented towards green development in traditional manufacturing enterprises, whose theoretical insights are mainly embodied in three aspects.

(1) In this study, the multi-case study method has reinforced the universality of the research findings, which in turn has compensated for the inadequacy of the fact that most of the existing studies on the business model innovation path of manufacturing are single case studies. The four core categories of efficiency orientation, value orientation, user orientation, and ecological orientation are derived to illustrate the differentiated green development orientation of manufacturing under the background of the digital economy and green development. Furthermore, the four green business model innovation paths are presented and compared in this foundation, which extends the relevant theories on the types and choices of green business model innovation paths. Moreover, it compensates for the inadequacies of the current relevant research, such as a single perspective and vague definition of paths.
(2) The relationship between the digital economy and green development has been deepened. This study combines the digital economy and green development and integrates their synergistic relationship into the business model, which is a further improvement of the relationship between the digital economy and green development discussed in the existing literature. Existing studies have shown that there is a two-way relationship between the digital economy and green development, but most of them are macro-oriented and lack consideration of the relationship between the two from the perspective of the business model. Through the case study, this paper finds that the business model innovation process under the guidance of green development of enterprises cannot be separated from digital technology, and the business model innovation under the digital economy can promote the sustainable development of the economy and reduce the related negative environmental externalities.
(3) This paper expands the theory of business model innovation. On the one hand, it enriches the connotation of business model innovation theory. Most of the existing research on business model innovation focuses on economic influence. This paper takes into account the challenges brought by environmental change and resource scarcity, emphasizes the important position of green development orientation in the process of business model innovation, and seeks business model innovation paths that take into account both Economic benefit and environmental efficiency. On the other hand, it expands the application situation of business model innovation theory. Based on the “China story” under the background of the digital economy and green development, this study takes a number of Chinese local manufacturing enterprises as typical cases to reveal the common experience of manufacturing enterprises’ business model innovation and provides specific path suggestions for Chinese manufacturing enterprises in the critical period of transformation how to achieve business model innovation.

### 7.2. Management Implications

Manufacturing enterprises need to choose an appropriate green business model innovation path based on their actual situation. This paper puts forward the following management implications for manufacturing enterprises to implement business model innovation.

(1) Enterprises with large-scale automation capabilities, comparatively mature and stable markets, and the desire to reduce costs and increase efficiency can opt for the efficiency-oriented path. Enterprises can use digital simulation, virtual reality, and other technologies to digitize difficult-to-materialize technologies and processes, automate production, and enable intelligent data analysis and application. In the innovation process, enterprises should pour attention to top-to-bottom strategic planning and introduce appropriate technologies based on their own development needs to avoid misunderstanding efficiency and only pursue advanced technology.

(2) Enterprises with core technology research and development capabilities, high technical barriers in the industry, and the desire to realize the upgrading of the industry value chain positioning can opt for the value-oriented path. Enterprises can develop digital services through digital technology and form a digital service platform to realize business value-added. In the innovation process, enterprises should focus on core technology development and service expansion and provide diversified, differentiated, and personalized integrated services for users. This approach avoids the frustration of transformation that resulted from the severe homogenization of industry products and services.

(3) Enterprises with products relevant to the daily lives of the general public and the desire to lock in consumer resources can opt for the user-oriented path. Enterprises can achieve close interaction with the demand side through digital platforms to realize user value co-creation so that users’ individual needs and emotional needs can be fully expressed. In the innovation process, enterprises should prioritize in-depth interaction with users, comprehend user needs through big data analysis, and organize production with a user-centric focus. Simultaneously, they should develop a flexible supply chain, alter their business processes, and enhance their adaptability to adjust to the complex environment of internal and external changes.

(4) Enterprises with core products with the foundation of embedded intelligent modules and the desire to extend product boundaries further can opt for the ecology-oriented path. Enterprises can produce intelligent products and form a business ecosystem through digital interconnection, gradually building a system of intelligent, interconnected products. In the process of innovation, enterprises should focus on the cohesion of smart product modules and inspire data sharing between products. At the same time, in the process of ecosystem construction, enterprises should consider products comprehensively to avoid the problems of product positioning and product management by excessively concentrated or scattered investment in the ecological chain.

### 7.3. Policy Implications

Based on the case analysis and findings, this paper puts forward the following suggestions on how to promote the green development of the manufacturing industry.

(1) Actively guide the manufacturing industry to implement innovative green business models. The government encourages enterprises to use market-based methods to achieve relevant green business model innovations, gives full play to the incentive role of fiscal policies, and reasonably amplifies the signaling role of government subsidies. For example, the government can provide substantial rewards and honorary support to enterprises that carry out green business model innovations, adjusts the ratio of ex-post to ex-ante subsidies, and explores diverse forms of subsidies and recognition.
(2) Give full consideration to the heterogeneity of enterprises and implement differentiated support policies. The competent authorities should implement differentiated innovation support policies according to the technical level of the industry, the attributes of the industry’s production, the attributes of the industry’s market, and the product types of the enterprise. For example, the steel industry can promote its automation and intelligent development, the consumer goods manufacturing industry can promote its personalized customization, and the high-end equipment manufacturing industry can promote its service-oriented transformation. By conducting pilot evaluations in specific industries, local conditions can be adapted.

## 8. Limitations and Prospects

First, the qualitative research method itself has some limitations. For example, there may be some inevitable subjectivity in the coding process, which needs to be empirically tested by large samples. Second, green development and technological innovation in the case show an interactive driving phenomenon. Hence, future studies may investigate the interaction mechanism between technological innovation and green development. Finally, digital change and green development concepts will continue to affect the path of business model innovation, thereby necessitating the tracking of new cases and practices in the future. 

## Figures and Tables

**Table 1:** Information on case companies.

Name	Address	Segmented Industry	Leading Products	Founding Time
Goldwind Science & Technology Co., Ltd.	Xinjiang, China	Wind turbine industry	Wind turbine manufacturing	1998
Baoshan Iron & Steel Group Co., Ltd.	Shanghai, China	Steel industry	Steel manufacturing	1992
Xiaomi Co., Ltd.	Beijing, China	Electronics industry	Electronics manufacturing	2010
QuMei Home Furnishings Group Co., Ltd	Beijing, China	Furniture industry	Furniture manufacturing	1987

**Table 2:** Specific sources of case information.

Material Sources	Source Examples	Source Marker
Public interviews, transcripts of speeches endorsed by executives	Senior executives are interviewed by leading magazines and speak on special occasions or at major events, such as anniversary celebrations.	Goldwind-I-GW Xiaomi-I-XM QuMei-I-QM Baosteel-I-BS
Authority obtained by official channels Information	Official website information, internal enterprise journals, financial report disclosure, and annual report of listed companies.	Goldwind-C-GW Xiaomi-C-XM QuMei-C-QM Baosteel-C-BS
Documentation	Literature downloaded from CNKI, Web of Science, etc.	Goldwind-D-GW Xiaomi-D-XM QuMei-D-QM Baosteel-D-BS
Research report	Research reports of brokerage institutions downloaded from databases, such as Wind and CSMAR.	Goldwind-R-GW Xiaomi-R-XM QuMei-R-QM Baosteel-R-BS
Publishing books	“Xiaomi philosophy Lei Jun ‘s business ecological operation logic ”; “Goldwind Science & Technology Innovation Road.”	Goldwind-B-GW Xiaomi-B-XM QuMei-B-QM Baosteel-B-BS
Other Internet channels	Materials obtained through news media and social platforms	Goldwind-E-GW Xiaomi-E-XM QuMei-E-QM Baosteel-E-BS

**Table 3:** Open coding results.

Subcategories	Conceptualization	Typical Source Material Examples
Need to reduce production costs (dd1) (d1,d2,d3)	Raw material cost increases d1	“The prices of raw materials such as iron ore, coal, alloy, and scrap steel have increased significantly, eroding the profits of the steel industry, which further tests our cost control ability.” (I-BS)
	Labor cost increases d2	“With the gradual disappearance of the demographic dividend in China and the improvement of the national social security policy, the labor cost keeps rising, which brings us new pressures and challenges” (I-BS)
	Steel prices decline d3	“Affected by repeated domestic epidemics, the domestic economy is weak, the pace of consumption recovery is not as good as expected, the demand of steel is declining, and the steel market is generally showing a weak demand and low expectations, and steel prices are low” (I-BS)
Need to produce high-quality products (dd2) (d5,d6)	Faced with overcapacity d5	“China’s steel industry is generally faced with the problem of” low-end surplus and high-end shortage ”of structural excess capacity” (R-BS)
	A big gap in the high-end product market d6	“There is still a big gap in the market of high-end products with higher added value, and we have shown insufficient adaptability and flexibility in dealing with high-quality product manufacturing and multi-variety production organization before.” (I-BS)
Need to save energy and reduce emissions (dd3) (d7)	Make energy saving and emission reduction target d7	“For the steel industry with high energy consumption, there is a long way to go to save energy and reduce emissions. We should have the technological capability to reduce carbon by 30% in 2025, strive to reduce carbon by 30% in 2035, and achieve the goal of "carbon neutrality" in 2050.” (I-BS)
Introduction of automated equipment (dd4) (d8,d9)	Accelerate the application of industrial robots d8	“Accelerate the application and promotion of industrial robots, double the number of robots in 2021, and keep the number of robots ahead of the industry.” (I-BS)
	Building an intelligent infrastructure d9	“We build a complete intelligent manufacturing infrastructure with intelligent equipment as the port” (I-BS)
Standardization of operational processes (dd5) (d10,d11)	Process management and optimization d10	“Process and system optimization is a work that is constantly updated. Therefore, a system support and operation team is set up within the sharing center, which is mainly responsible for sorting out which processes need to be optimized and organizing the establishment of optimization projects” (I-BS)
	Standardized process d11	“Because of the long process characteristics of Baosteel’s internal management, the importance of process management and optimization was fully emphasized at the beginning of the establishment of the sharing center, and the process was straightened out and standardized, which laid the foundation for subsequent stability.” (I-BS)
Basic data collection (dd6) (d12)	Real-time data collection and integration d12	“The real-time data of thousands of monitoring devices installed in Baosteel’s production site, including various instruments, thermocouples and other monitoring devices, are collected into the process control computer system through the field bus.” (I-BS)
Data integration (dd7) (d13,d14,d15)	Unified data standard d13	“We improve system flexibility by adopting unified data standards” (I-BS)
	Achieve unit collaboration d14	“By adopting different deployment methods, the information systems of the group management and control and the main iron and steel business management layer can quickly cover new units, strengthening the group management and control, and strengthening the collaboration between new units and other units.” (I-BS)
	Connect data node d15	“Cut through the steel manufacturing application scenario, connect all edge data nodes, and realize more comprehensive intelligent management and control of steel mill processes and production lines” (I-BS)
Production automation (dd8) (d16,d17,d18)	Unmanned production d16	“Through the application of new technologies such as automation, information technology and artificial intelligence, unmanned driving is realized, and unmanned logistics operations such as reservoir management and steel coil lightering are realized; Dirty and dangerous positions such as unit entrance baling, zinc pot slag fishing, export sampling, baling and labeling are unmanned.” (C-BS)
	Automation of equipment and process control d17	“By promoting the unmanned scene, accelerating the application of new technologies such as industrial robots, unmanned driving, artificial intelligence, and building a steel product production line, the automation of process equipment and process control has been completed. Shanghai base is the first in the world to realize one-button steelmaking and tapping.” (I-BS)
	Construction of fully automated production line d18	“Under the company’s “four unifications ”intelligent manufacturing plan, the hot rolling mill has started a new round of “1+N” full-automatic production line construction featuring automation, intensification and digitalization, and the transportation chain, rough rolling and coiling areas have been fully automatic.” (C-BS)
State perception (dd9) (d19,d20)	Real-time monitoring of equipment status d19	“Preliminary completion of three-dimensional construction simulation of key equipment maintenance projects, real-time monitoring of equipment status, intelligent diagnosis of equipment faults, and practical improvement of work efficiency and quality of equipment maintenance and fault diagnosis.” (I-BS)
	Product quality inspection d20	“We apply the steel surface defect detector, based on machine vision and image processing technology, to detect all kinds of surface quality defects of steel online in real-time.” (I-BS)
Intelligent decision-making (dd10) (d21)	Establish an intelligent grading decision system d21	“Apply artificial intelligence machine learning and deep learning model to improve the defect detection and classification accuracy of strip surface defect detector, establish an intelligent grading decision system for strip surface defects, replace manual quality inspection, and realize automatic grading and intelligent management of strip surface quality” (C-BS)
Advance warning (dd11) (d22)	Anticipate business risks and give an early warning d22	“Steel is a large-scale commodity, with a large transaction amount and high risk. We comprehensively analyze and evaluate the credit level and debt-paying ability of our customers by using the internal and external market, operation and legal data, so as to predict the business risk of the enterprise as early as possible and issue an early warning of trading behavior risk” (I-BS)
Intelligent visualization (dd12) (d23)	Visual Digital Twin Application Scene d23	“Through the data interaction of 3D model, simulation model, and equipment condition monitoring model, we complete digital mapping of virtual space and realize the integration of static data of engineering construction and real-time dynamic data of production and operation to form a visual digital twin application scene ” (I-BS)
Dynamic and accurate execution (dd13) (d24,d25)	The intelligent temper mill control model d24	“Establish an intelligent temper mill control model to achieve automatic optimal matching control of elongation, rolling force, surface quality and shape.” (I-BS)
	Accurately control the temperature d25	“Through artificial intelligence virtual measurement technology, the temperature of strip steel in zinc pot can be accurately controlled, and the surface quality of strip steel can be significantly improved.” (I-BS)
Data center platform (dd14) (d26)	Building a Big Data Center d26	“We started the construction of a smart manufacturing big data center, which is intended to plan the evolution of a new generation of information architecture. Based on the cloud data nodes, a business middle station that controls the whole process of steel manufacturing services and a middle data station that provides intelligent decision-making ”(I-BS) will be formed.
Data analysis and application (dd15) (d27)	A digital ecosystem of application interconnection d27	“The big data center with the new architecture will completely break the “information island,” realize data co-acquisition, data sharing, system co-construction and function sharing, create a digital ecosystem of application interconnection, and further penetrate the whole process of real-time data collection, analysis and decision-making.” (I-BS)
Need to expand sustainable value business (dd16) (d28,d29,d30)	Change of profit point of wind power industry value chain d28	“The strategic control points and profit points of the wind power industry value chain have changed with the expansion and change of market space. The manufacturing and sales of wind turbines, which once had high profits, have resulted in overcapacity, profit compression, or even disappearance due to the swarming of competitors, resulting in low-price competition in the market.” (I-GW)
	Expand energy environmental protection business d29	“We stick to the business development route that is beneficial to the society and the environment while consolidating the wind power manufacturing business, we actively expand the energy conservation and environmental protection business for our customers and contribute to the sustainable development of the environment and society.” (I-GW)
	Providing solution business d30	“Solving problems and providing solution business to make the clean energy industry cleaner is not only the requirement of national green development, but also a major development trend and new growth opportunity of the industry.” (I-GW)
Need to enhance the value proposition (dd17) (d31,d32)	Remodeling Enterprise Value Positioning d31	“Goldwind Technology has established the strategic positioning of the overall wind power solution provider. Strive to change from a single product manufacturer to a system solution provider and system service provider in the field of energy conversion, from product management to brand management and capital operation.” (I-GW)
	Help more enterprises reduce carbon emissions d32	“While continuing to carry out scientific and technological innovation, we will further explore zero-carbon products and solutions with innovation as the engine. In the future, we will help more enterprises reduce carbon emissions and energy use costs by deploying green energy.” (I-GW)
Need to create higher value space for customers (dd18) (d33)	Create more value for customers d33	“We pursue high quality, high reliability and high power generation efficiency instead of being big. We constantly optimize the overall solution and strive to create more valuable space for customers in every link of wind power development and operation. Strive to create more value for customers by improving the quality of products and services ”(I-GW)
Core technology R&D capability (dd19) (d34,d35)	Continuous scientific and technological innovation d34 Independent research and development d35	“Our continuous investment in scientific research and innovation has solved the green development problem of the company. In the end, we have obtained independent intellectual property rights, which has established the company’s leading position in the world.” (I-GW)-d34 “Our self-developed direct-drive permanent magnet technology has laid a commanding lead for long-term sustainable development, and Goldwind’s wind turbine technology products can be said to have been in the leading position in the world.” (I-GW)-d35
Data collection and analysis capabilities (dd20) (d36)	Abundant data d36	“Since its establishment, we have attached great importance to the collection and collation of basic data. Since the establishment of the company, we have established basic databases such as business database and financial database, and mastered abundant basic data.” (I-GW)
Specialized service capability (dd21) (d37,d38)	Accumulate management and technical experience d37 Stable customer relationship d38	“The company has accumulated rich experience in the development of high-quality wind resources and the operation and management of wind farms(d37). At the same time, it has the leading technology in the wind power industry. Through years of mature wind turbine sales, our capital chain customer relationship network has matured(d38)” (I-GW)
Additional Services for Products (dd22) (d39,d40,d41,d42)	Product maintenance d39 Product upgrades d40 Product logistics management d41 Product development and design d42	“Goldwind Technology continues to provide warranty service at an additional charge; Wind farm operation and maintenance business, that is, Goldwind Technology helps users manage wind farm assets; Other separately paid services, including customer customization, sales of spare parts, technical consultation and technical services, technical transformation and upgrading of wind farms, maintenance of wind turbines, logistics management” (C-GW)
Engineering-procurement-construction (dd23) (d43)	Signing EPC general contract d43	“We signed the EPC general contract with Shanghai Institute in a consortium way, and jointly undertook all the work from the preliminary design of the project to the overall completion acceptance, and built the Dafeng H8-2 project into a new model of an offshore wind farm.” (I-GW)
Solution services (dd24) (d44)	Provide smart energy solutions d44	“Taking the successful practice of Beijing Yizhuang Carbon Neutral Park as a starting point, we provide smart energy solutions for many large-scale conferences, scientific research centers, industrial parks, and ports, increase the proportion of green power use, and explore the creation of a new path of “zero carbon” development.” (I-GW)
Remote operation and maintenance services (dd25) (d45)	Provide remote operation and maintenance service d45	“In Australia and North America, where our international expansion is relatively mature, different business models bring opportunities to the operation and maintenance business. We cooperated with our tax investors Citibank and Berkshire Hathaway Energy Company on the Rattlesnake wind farm project in Texas. Besides supplying wind turbines, we also contracted all the remote operation and maintenance services of wind farms on an annual basis.” (I-GW)
Industrial financial services (dd26) (d46,d47)	Providing financial solutions and asset management d46 Financial Services in the Whole Industry Chain d47	“In the new era of wind power, we provide financial services in the whole industry chain, such as financial solutions and asset management, to realize the full ecological asset operation and value-added of wind power products. Goldwind Technology’s whole industry chain financial solution takes credit transmission and risk control as the core to improve the financial ecology of the industry.” (I-GW)
Cloud service platform (dd27) (d48,d49)	Operation platform d48	“We embedded wind power big data, intelligent operation and maintenance into wind power equipment manufacturing and overall wind power solutions and proposed a wind power intelligent operation 2.0 platform solution. The platform integrates SES and SM, and relies on the global monitoring service center set up in Beijing to provide more than 20,000 units with system integration operation and maintenance solutions” (I-GW)
	Digital Energy and Carbon Management Platform d49	“The “three carbon reductions plus one platform” created by Goldwind Technology, through the digital energy and carbon management platform, reduces carbon on the energy consumption side, the energy supply side and the transaction side, realizes the energy management and carbon emission management throughout the whole process, and provides the best economic path for enterprises to build carbon emission reduction.” (I-GW)
Need to increase the viscosity of users (dd28) (d50)	Maintain consumer brand loyalty d50	“For the home furnishing industry, where product sales have always been low frequency, low repurchase and high unit price, after-sales service and word-of-mouth maintenance have become the top priority. We always adhere to the principle of honesty first and formulate systematic and scientific marketing communication strategies, putting the real product quality and service level at the core, and with excellent product quality and service, creating the brand image of a good home for the people.” (I-QM)
Need to meet diversified market demands (dd29) (d51,d52,d53)	Catering to personalized consumption demand d51 Analysis of Ideal Lifestyle d52 Observe the details of life d53	“When the 80s and 90s are the main consumers, as brands, we should not only cater to the consumption needs of youth, individuality and fashion, but also be the “intimate friends” of consumers, not only knowing their decoration preferences, but also analyzing their lifestyle and understanding the true psychology of young people. We should know their ideal life better than consumers, observe the details of life, and provide meticulous and thoughtful service.” (I-QM)
Need to form a differentiation brand (dd30) (d54)	Trying to solve the problem of sales terminal differentiation d54	“In recent years, the furniture industry has entered the “homogenization” competition, and the products, prices, brand-building behaviors, store decoration, and display are increasingly consistent. We form the terminal “marketing potential energy”, raise the threshold of market competition, try to solve the problem of sales terminal differentiation, establish the added value of products, turn products into works, let consumers know Qumei in an environment, and create the market barriers of Qumei sales terminal.” (I-QM)
Big data analysis (dd31) (d55)	Big data analysis to accurately grasp consumer preferences d55	“We have reached in-depth cooperation with JD.COM, and with the help of JD.COM’s intelligent big data analysis capabilities, we have designed and laid out products around young consumers’ preferences.” (I-QM)
Personalized customization (dd32) (d56)	All products are open and customized d56	“All our product lines are open and customized, which can fully meet the individual needs of consumers.” (I-QM)
Flexible manufacturing model (dd33) (d57)	Flexible production chain and intelligent production workshop d57	“At present, Qumei Home, with its flexible production chain and intelligent production workshop, uses an ERP system to break the information island within the enterprise and realize intelligent collaboration, thus realizing the industry-leading mass customization mode.” (C-QM)
Create the brand characteristic scenario (dd34) (d58)	Convey the brand idea of the store d58	“We hired a well-known domestic design agency to open the 4th generation independent store in the Asian Games Village, Datun Road, Chaoyang District, Beijing, upgrading from a furniture brand to a fashion home brand. The independent store with the brand-new image of “Wan Ziwen” conveys Qumei’s aesthetic concept of “simplicity, elegance and refinement”, which has become the store image of Qumei that continues to this day.” (I-QM)
Experiential life scenario (dd35) (d59)	Establish an experiential life scene d59	“We break the one-dimensional relationship between products and consumers through the scene display design of stores, establish an experiential life scene, and complete the deep connection with consumers. In Qumei, consumers can not only see the unique home model rooms but also sit down to have coffee, read books, chat and have a deep experience of life scenes.” (I-QM)
Setting VR virtual interaction scenarios (dd36) (d60)	VR Experience Zone d60	“We have VR experience areas with different themes and styles in the comprehensive life experience store, so that consumers can feel the ideal home space.” (I-QM)
Online and offline consumption channels (dd37) (d61)	Online and offline mode d61	“Qumei’s home market network is extensive, and stores have been opened in more than 200 cities across the country. The “cement+mouse” marketing model pioneered by the industry perfectly integrates online shopping malls and physical storefronts and achieves a double leap in brand marketing power and market share. Qumei Home’s innovative channel model has created a new 24 h online shopping experience and a creative e-color space for consumers.” (C-QM)
Online and offline data integration (dd38) (d62)	Online and offline data fusion d62	“Our knowledge of offline users is limited to the “physical level”, such as how big a room they live in, how many people they have, while online users’ insights outline richer and more three-dimensional “portraits”, learn about consumers’ shopping categories and habits about home in JD.COM Mall, and directly guide the combination of goods on this basis.” (I-QM)
Establish a we-media system (dd39) (d63,d64,d65)	Social platform d63 Digital marketing d64 live streaming d65	“In order to quickly build the private domain traffic pool of the brand and broaden the marketing channels, we have continuously developed social platforms where young people such as WeChat official account, Weibo, Toutiaohao, Tik Tok, Auto Quicker and Xiaohongshu gather, and built Qumei self-media system with “fashion, richness, and value” as the keyword; Promote the normalization of online sales and live streaming sales with goods, and will continue to focus on online drainage and offline empowerment. Through more than 1000 stores in more than 400 cities across China, we will continue to lead people to visit stores, understand products and provide services by live broadcast.” (I-QM)
Digital stores (dd40) (d66,d67)	Digital store d66 Unbounded retail d67	“Before digitalization, offline stores had a limited understanding of “field”. Basically, they had to contact consumers and generate sales in the fastest way in a tangible and limited space. In the process of internet plus, intelligent, technological and experiential transformation, with the blessing of the camera, Wi-Fi probe and other store technologies, the “field” becomes more unbounded, the value chain becomes longer and longer, and it is easier for consumers to immerse themselves in “one-stop experience consumption”.” (I-QM)
Cross-border fusion scenario (dd41) (d68)	Cross-border fusion d68	“With Qumei Furniture as the logical center, different goods such as furniture, home and life are integrated. The close consumption relationship of categories in the home scene makes the consumption structure more and more diversified.” (I-QM)
Develop a brand-interactive community (dd42) (d69)	Launching the Wanzhong Design Platform d69	“We have launched the Wanzhong Design Platform, which provides users with an interactive platform to print pictures and communicate, and at the same time allows users to participate in home space design.” (I-QM)
Launch a user design platform (dd43) (d70)	Develop a 3D customized ecommerce platform d70	“We have developed a 3D customized e-commerce platform, with customization as the core concept. Customers can build their own floor plan on the 3D stereo software, place all kinds of furniture virtually according to their own wishes, and simulate their own reality to the greatest extent through parameter setting, so as to realize the free customization of the color and size of their own furniture.” (I-QM)
Need to achieve openness and cooperation (dd44) (d71)	Broad alliance d71	“The most important thing for our company is to form a broad alliance and cooperate with an open mind. The more friends, the better.” (I-XM)
Need to construct a business ecosystem (dd45) (d72)	Make the Xiaomi Ecosphere Goal d72	“We have set the goal of investing in 100 eco-chain enterprises within five years, forming a “Xiaomi Ecosphere” with Xiaomi Company as the core.” (I-XM)
Need to expand the enterprise boundary (dd46) (d73)	Not satisfied to only make the mobile phone d73.	“Xiaomi’s mobile phone brand has gradually become a climate and has a certain right to speak in the industrial chain. However, a millet that only makes mobile phones will not have a real future, and its effect will soon be suppressed. We need to get rid of the excessive dependence on mobile phone categories ”(I-XM)
Human–machine intelligent interaction (dd47) (d74,d75,d76)	Voice interaction d74	“The interactive mode has been upgraded from traditional buttons and touch screens to sounds, actions and other ways that are closer to human interaction.” (I-XM)
	Action interaction d75	“We also provide intelligent voice assistant Xiaoai students for Mijia products, and Xiaoai students provide services to users in various scenes in a more human-friendly way.” (I-XM)
	Intelligent voice assistant d76	“As an important terminal, the car’s interaction mode is very important. We cooperate with Li ONE Automobile to create a voice interaction environment in the car through Xiaoai classmates.” (I-XM)
Intelligent environment detection (dd48) (d77)	Monitoring environmental value d77	“We have introduced an intelligent maintenance product-Flower Monitor, which is the first product to “establish communication” between plants and people. It can help flower lovers to better raise flowers and enhance the maintenance pleasure by monitoring the values of temperature, moisture, fertility and light of green plants.” (I-XM)
R&D intelligent modules (dd49) (d78)	R&D intelligent modules d78	“We invest in Green Rice, specializing in the research and development of intelligent modules for smart homes. By embedding this module, home appliances become intelligent, and through sensors, we can find out whether users are home or not, thus providing related services, which attracts users to use Xiaomi products.” (I-XM)
Intelligent analysis function (dd50) (d79)	Provide exercise plan through intelligent analysis d79	The acceleration sensor in mi band measures the change of direction and acceleration to count the steps, and processes the data with intelligent computing ability to match the user’s exercise type, and then monitors the user’s walking number and calorie consumption, providing data support for the user to make a more scientific exercise plan. (C-XM)
Ecological-chain pattern (dd51) (d80,d81)	Set up ecological chain department d80 Extended product d81	“We set up an eco-chain department, which extends to mobile phone peripherals, intelligent hardware and daily consumables products through the mode of ‘investment+incubation’ eco-chain enterprises. ”(I-XM)
Collaborative research and development (dd52) (d82)	Cooperation in research and development d82	Xiaomi also cooperated with all seasons hotels, cars, homes and love spaces, committed to research and development innovation in the field of AloT, and launched the developer incentive plan-Xiaomi AIoT Developer Fund at the conference, with an initial investment of 100 million yuan. (C-XM)
Cross-border integration (dd53) (d83)	Cross-border integration d83	“We have reached cross-border integration with IKEA, a home furnishing company, and all intelligent lighting products of IKEA will be connected to Xiaomi IoT platform … Xiaomi and IKEA have a high degree of agreement, and this cooperation will accelerate the intelligentization of the global home furnishing industry” (I-XM)
Resource sharing (dd54) (d84)	Share common resource d84	“The purpose of Xiaomi’s ecological chain is to share common resources and invest in innovative enterprises to deepen the segmentation. We focus on the main framework, such as design and quality, which is specifically produced by eco-chain companies” (I-XM)
Ecological platform construction (dd55) (d85)	Platform strategy d85	“The strategy of the Internet of Things is not to be hardware, but to be a platform. Apply standards to existing hardware vendors through the platform portal” (I-XM)
	Online ecological cloud platform d86	“We launched the eco-cloud platform, so that enterprises can not only store data in the cloud, but also make products share the data in the cloud, breaking product boundaries and further improving product functions” (I-XM)
Data standardization (dd56) (d87)	Unified chip d87	“In the process of expanding intelligent hardware, we found that some product intelligent modules are not unified, and data cannot be integrated. We rely on Songguo Electronics to develop a unified IoT smart chip, and promote products to generate standardized data and realize interactive linkage.” (I-XM)
Unified product style (dd57) (d88)	Harmonized product style d88	“In the Internet of Things era, many products serve the same user, and the user consistency experience is crucial. Xiaomi has hundreds of products, which are unified and coordinated, with a wide variety but not messy. This is due to Xiaomi’s minimalist design principle, which not only reduces the difficulty of the production line, but also ensures that all product styles are coordinated and unified.” (I-XM)
Scenario linkage (dd58) (d89)	Connect between scenes d89	“It’s very simple. As long as we master the data entry and application, we can apply user and product data to various fields simultaneously, and promote the connection between scenes. For example, embedding Xiaoai classmates into IKEA lighting system to expand To B fields such as hotels, travel and real estate, which will bring immeasurable value.” (I-XM)
Providing life scenario system solutions (dd59) (d90)	A life scene system solution d90	“We provide systematic solutions for users. For example, Xiaomi Eco-chain enterprises embed chips in bracelets and lamps respectively, and jointly launch a “quality sleep solution”, that is, the lamps will automatically adjust the brightness according to the user’s sleep state detected by mi band.” (I-XM)
Product function interaction (dd60) (d91)	Interaction between products d91	“For example, Dr. Xiaomi Eco-Chain Baby’s toothbrush, toothbrush and small love speaker can interact with each other, and the story will be automatically played when children brush their teeth, which can guarantee the user’s brushing time and provide a value-added experience for the product.” (I-XM)
R&D connected modules (dd61) (d92)	Release connected modules d92	“We released the Wi-Fi module, which was only priced at 9.9 yuan, which greatly reduced the cost of the Internet of Everything. We will open the platform for third-party brands, and products can access the loT platform as long as they are embedded in this module, which expands Xiaomi loT Scope.” (I-XM)

**Table 4:** Axial coding results.

Main Category	Subcategories	Numbering
Efficiency Orientation	Need to produce high-quality products; need to save energy and reduce emissions; need to reduce production costs	D1
Value Orientation	Need to expand sustainable value business; need to enhance the value proposition; need to create higher value space for consumers	D2
User Orientation	Need to increase the viscosity of users; need to meet diversified market demands; need to form a differentiation brand	D3
Ecology Orientation	Need to achieve openness and cooperation; need to construct a business ecosystem; need to expand the enterprise boundary	D4
Production Process Automation	Introduction of automated equipment; standardization of operational processes; basic data collection; data integration; production automation	D5
Core Competence Shaping	Core technology R&D capability; data collection and analysis capabilities; specialized service capability	D6
Product Intelligence	R&D intelligent modules; human–machine intelligent interaction; intelligent environment detection; intelligent analysis function	D7
Intelligent Transformation	Data center platform; state perception; intelligent decision-making; advance warning; intelligent visualization; dynamic and accurate execution; intelligent data analysis and application	D8
Servitization Transformation	Engineering-procurement-construction; solution services; remote operation and maintenance services; industrial financial services; cloud service platform	D9
Ecological Synergy	Ecological-chain pattern; ecological platform construction; cross-border integration; collaborative research and development; resource sharing	D10
Smart Connected Product Systems	R&D connected modules; data standardization; product function interaction; unified product style; providing life scenario system solutions; scenario linkage	D12
Personalized Manufacturing	Big data analysis; personalized customization; flexible manufacturing model	D13
Scenario Innovation	Create the brand characteristic scenario; setting VR virtual interaction scenarios; digital stores; experiential life scenario; cross-border fusion scenario	D14
User Participation	Establish a we-media system; launch a user design platform; develop a brand interactive community	D15
Additional Services for Products	Product maintenance; product upgrades; product logistics management; product development and design	D16
Online and Offline Integration	Online and offline consumption channels; online and offline data integration	D17

**Table 5:** Selective coding results.

Path	Category
Efficiency-oriented path	Efficiency orientation; production process automation; intelligent transformation
Value-oriented Path	Value orientation; core competence shaping; additional services for products; servitization transformation
User-oriented path	User orientation; user participation; personalized manufacturing; scenario innovation; online and offline integration
Ecology-oriented path	Ecology orientation; ecological synergy; product intelligence; smart connected product systems

**Table 6:** Theoretical saturation test results.

Category	Case Company	Examples of Typical Data
Efficiency orientation; production process automation; intelligent transformation;user orientation; user participation; personalized manufacturing;	China Chang’an Automobile Group Co., Ltd., Beijing, China.	“We focus on how to achieve high-quality product output, reduce inventory turnover and reduce the cost of complete vehicle manufacturing.”-efficiency orientation “We introduced industrial robots, improved our technology level, set up our own automated production lines and improved economies of scale.”-production process automation “We use simulations to verify vehicle safety and system stability. Later, the IT system architecture on Ali Cloud greatly reduced the deployment time and cost. Through external integration ability and structural flexibility, digital technology is applied to the whole process of product production and sales, and finally realize the intelligent process.”-intelligent transformation “Changan Automobile adheres to the product orientation of “customer first”, and through big data analysis of customer behavior patterns and basic information, excavates customers’ explicit and potential demands, and continuously creates value for customers.”-user orientation “We let users participate in the whole process from product research and development to the final product sales and pricing. We emphasize user feelings and attach importance to the expression of the emotional value of products and services, forming a set of design standards and manufacturing standards for customer needs.”-user participation “Our R&D brain platform can automatically match and recommend modular product integration according to user-set parameters”-personalized manufacturing …
Value orientation; core competence shaping; additional services for products; servitization transformation	Shenyang Machine Tool Co., Ltd., Shenyang, China.	“The era of low profit in China’s machine tool industry has arrived. The machine tool market continues to be depressed, and the market competition is more fierce. It is necessary to expand high value-added business”-value orientation “By reforming the system, reorganizing the departments, reforming the process and strengthening the management, we have effectively realized the reform of the enterprise management mode, promoted the enterprise’s learning and absorption of external knowledge, and completed the learning and absorption of the introduced technology, so as to improve our technical level.”-core competence shaping “We provide services based on customer demand characteristics such as quality, service, delivery time and price. At this stage, our focus is still on manufacturing, and passive services such as basic auxiliary installation and after-sales maintenance are only set as part of additional product attributes to improve product sales.”-additional services for products “Through innovation and systematic adjustment, the company has realized the transformation of profit model, realized the magnificent turn from machine tool manufacturer to the industrial service provider, and carried out the strategic transformation from industrial manufacturer to intelligent manufacturing comprehensive solution provider.”-servitization transformation …
User orientation; user participation; personalized manufacturing; online and offline integration	Qingdao Red Collar Group Co., Ltd., Qingdao, Shandong, China	“Outstanding personalized needs are increasingly being paid attention to by merchants. In the garment manufacturing industry, consumers pay more attention to personalized needs, which provides fertile soil for Red Collar’s business model. Red Collar Group is driven by consumer demand.”-user orientation “In order to better interact with users, we build a virtual and real interaction platform for users through the Internet”-user participation “The original “three-point-one-line” volumetric method collects 24 data from 19 parts of the body and inputs them into the Red collar personalized customization platform. The system will conduct data modeling and quickly form a data version exclusive to the customer.”-personalized manufacturing “This is the integration of online and offline, offline to complete the experience, see the appearance of the garment, measure the figure, introduce the category and collocation of clothes, and then enter the online customization. Also through online activities a large number of diversion, offline conversion, further reducing marketing costs.”-online and offline integration …
Ecology orientation; ecological synergy; product intelligence; smart connected product systems	Haier Group Co., Ltd., Qingdao, Shandong, China	“Constantly introduce new products, new models and new business forms, constantly break out new ecosystems, and realize the global layout of multi-brand collaboration”-ecology orientation “Haier COSMO platform has gathered more than 300 million users and more than 3.8 million global ecological resources, realizing cross-industry and cross-field expansion and service.”-ecological synergy “We enable products to hear, speak and think, to achieve the natural interaction between hardware products and users, and to achieve the best experience for users”-product intelligence “We realize the real-time interaction of platform hardware and software devices, use artificial intelligence to realize the interconnection between people and things, and finally use the ecosystem to provide users with intelligent interconnected products, and quickly show off their talents in the Internet of Things smart home appliances.”-smart connected product systems …
Value orientation; core competence shaping; additional services for products; servitization transformation	Xi’an Shaangu Power Co., Ltd., Xi’an, China.	“With the increasingly fierce market competition, the exploration of high value-added areas of the industrial chain is related to the survival of enterprises and the fundamental interests of employees.”-value orientation “We get out of the single domain of turbine machinery thinking and continue to process Reengineering, resource integration, optimization of support system and other measures, from the operation, equipment, Service and other dimensions to comprehensively improve our technical level”-core competence shaping “Provide maintenance, remote monitoring, zero inventory of spare parts, equipment upgrading and other supporting basic support services for the safety and efficiency problems existing in the operation of energy conversion equipment”-additional services for products “Based on customers’ functional requirements for energy conversion equipment, provide integrated operation services of the integrated park and operation and maintenance services for taking over customers’ operation activities such as electricity, gas and solid waste treatment”-servitization transformation …

**Table 7:** Comparison of business model innovation paths.

	Path Type	Efficiency-Oriented Path	Value-Oriented Path	User-Oriented Path	Ecology-Oriented Path
Contents					
Starting point		Start on production process automation	Start on developing core technologies	Start on user social platform design	Start on the transformation in product intelligence
Direction and focus		Follow the direction of improving production efficiency. Achieve intelligent manufacturing, improve the efficiency of resource utilization, and drive enterprises to upgrade to high-end products.	Follow the direction of enhancing the value by servitization. Shape the advantages with core capability, achieve the service-oriented extension, and provide high-value and unique services.	Follow the direction of improving user relationships. Implement personalized manufacturing, scenario innovation, and online and offline integration to improve the consumer experience.	Follow the direction of expanding the enterprise boundary. Build smart connected product systems through the “Internet of Everything” to achieve resource sharing across organizational boundaries.
Process		Digitalization of the production process ←→ automation of the production process → intelligent transformation	Core competence shaping → additional services for products → servitization transformation	User participation ←→ flexible manufacturing → personalized manufacturing ←→ scenario innovation → online and offline integration	Product intelligence → product interconnection → smart connected product systems based on the ecological platform
Environmental efficiency		Production automation and intelligence can increase the efficiency of resources and energy, achieve energy saving and emission reduction in the production process, and get rid of overcapacity dilemma.	Service is an important intangible resource, and the enhancement of service alleviates the consumption of product resources and provides sustainable value for industry development.	Precise, personalized manufacturing overcomes product homogeneity and prevents resource mismatch and waste. Scene innovation pursues the comparison of user experience and avoids the vicious competition of resources.	Intelligent products have stronger performance and higher utilization. Through the business ecosystem, we can share resources, knowledge, wealth, and value creation, improve the value of resource allocation and realize the value of resource combination.
Economic benefit		Production automation and intelligence can not only improve output but also reduce production costs and achieve economies of scale. It also helps to improve product quality and open up the high-end market.	Servitization in manufacturing is the main value-added point. Unique digital services create a core competitive advantage and expand profit channels, stimulating service consumption to foster new economic growth points.	Personalized customization brings product premium and forms differentiated products to increase the viscosity of users. Scene innovation can shape a unique brand concept to encourage users to consume.	The intelligent connected product system expands the industry boundaries and product categories, improves the added value and function of products, and drives the supporting consumption of ecological products.
Innovation risk		The innovation process is prone to a shortage of human resources, resulting in the introduction of a large number of automatic equipment and technologies but not their efficient application.	The development of servitization is a long-term process with a high risk of domain expansion and requires core technology and service capabilities as support.	Personalized manufacturing may lose mass-production economies of scale and requires sustainable production methods. Scenario innovation necessitates continuous renewal.	The expansion of the ecosystem may bring about data silos, collaboration difficulties, and product connectivity problems, which prevent further value-added data and result in ecological disintegration.
Applicable condition		The industry has a certain degree of technological absorption and large-scale automation capability, and the market is relatively mature and stable.	Possess core technology research and development capabilities and original innovation capabilities. Presence of high technical barriers and long value chains	The products are close to the daily lives of the public. Enterprises have their brands and many marketing and sales channels.	The core products have been successfully built. Products have the basis for embedded smart modules. Enterprises are able to share resources with suitable partners.
Applicable enterprise		Steel, chemical, and other process manufacturing. Discrete manufacturing with a high degree of modularization	Producing highly technical and complex products and facing enterprise-level consumers, such as equipment manufacturing, new energy enterprises	Products have low technical barriers and are aimed at mass consumers, such as furniture, food, and clothing.	High-technology industries in consumer goods manufacturing, such as automotive, home appliances, and electronic equipment.

## Data Availability

The datasets used or analyzed in this are accessible from the corresponding author on demand.
